# STAT: schema therapy for addiction treatment, a proposal for the integrative treatment of addictive disorders

**DOI:** 10.3389/fpsyg.2024.1366617

**Published:** 2024-07-02

**Authors:** Elizabeth Lacy

**Affiliations:** Elizabeth Lacy, LCSW, PLLC, New York City, NY, United States

**Keywords:** addiction, attachment, personality, schema, schema mode model, holistic, treatment, detached protector

## Abstract

The nature and origins of addictions and of their adjunctive behaviors, as well as their chronicity, call for treatments that conceptualize and treat them as the long-term and complex processes that they are. Addictions are often comorbid with personality problems and with trauma histories. Patients suffering from these disorders often show poor engagement with treatment and high rates of relapse, possibly because available treatments have yet to address the patient suffering from addiction in a more integrated or holistic manner. In particular, comprehensive treatment models for addictive disorders – like treatments for personality disorders or trauma – are likely to require the integration of behavioral, cognitive, *and* emotion-focused interventions within a facilitative therapeutic relationship. However, most current treatment models, including ones which are highly effective in stabilization or behavioral change, lack one or more components of treatment that could sustain longer term recovery, wellness, and health for a higher percentage of patients. In this article, I propose approaching addictions and their treatment from the perspective of schema therapy, an integrative, developmental model with a strong track record of positive outcomes in addressing personality disorder symptoms and long-standing trauma histories, commonly comorbid with addictive disorders. In advancing this proposal, I begin by providing some background tying together addictions, attachment, and personality, suggesting they be treated simultaneously to achieve improved outcomes. Then, after briefly reviewing the leading approaches to the treatment of addictions, I introduce the idea that schema therapy is well-situated – both theoretically and practically – to address many of the shortcomings of existing treatment options. In particular, I note how addictive and co-occurring colluding behaviors are deeply intertwined with both early and continued frustration of core developmental needs. I illustrate how the addictive cycle is perpetuated through the process of schema reinforcement and through the operation of schema modes. I then demonstrate how these key terms (i.e., needs, schemas, and modes) inform the patient’s assessment and case formulation, guiding treatment interventions from a strong therapeutic relationship that focuses on integrating recovery behavior change, healing dysfunctional schemas and modes, and preventing relapse.

## 1 Introduction

Addictive disorders are intrinsically difficult to treat for several reasons. First, individuals with such disorders are often cross-addicted (e.g., chem-sex addictions; Carnes et al., 2005; Sussman et al., 2011). Second, addictive disorders are highly comorbid with personality disorders (PD) (e.g., Sher et al., 2000; Kienast et al., 2014; Boog et al., 2018), with rates of full diagnostic criteria for PD diagnoses estimated to be as high as 60–65% among patients suffering from addictions (Casadio et al., 2014; Roncero et al., 2019). Such comorbidity is tied to poorer outcomes in the treatment of most disorders (Ball, 1998; Fatseas et al., 2015) including addictions (Padykula and Conklin, 2010; Brorson et al., 2013). Third (and relatedly), the etiology of addictive behaviors (as well as of personality disorders) has been linked to early childhood trauma and neglect and is often associated with poor attachment experiences and subsequent difficulties with emotion regulation (Hektner, 1997; Flores, 2001; Crowell et al., 2009).

Attachment is a highly dynamic, neurobiological process, entwined with temperament, which helps the developing child, adolescent, and adult regulate negative affect and facilitates healthy functional relationships (Cassidy, 1994; Estévez et al., 2017; Mikulincer and Shaver, 2019). Inadequate attachment, which may stem from a variety of personal and environmental variables such as absent or distracted parents, abuse, neglect, poverty, illness, and the like, makes it difficult for the infant or developing child to sooth emotional pain (Rees, 2007). Eventually, an inadequately attached individual must find methods other than attachment to caregivers to tolerate painful feelings (Hoeksma et al., 2004; Miljkovitch et al., 2018). At times, this involves turning to highly engaging behaviors which provide rapid gratification – a pattern which may later develop into addictive disorders (Aldao et al., 2010; Burkett and Young, 2012).

People suffering from addictive disorders often carry with them the experience of childhoods full of escapist attempts at emotion regulation (Betts et al, 2009). These attempts may include retreating to fantasy worlds while parents fought (Estévez et al., 2017), playing video games in place of socializing with friends, or engaging in fantasies about feeling loved or important after (and sometimes during) abusive episodes. As these children become adolescents, they often gain access to even more expedient and/or effective avoidance strategies, such as illicit substances, masturbation, shopping, and gaming (Burkett and Young, 2012). Indeed, some addictive behaviors, particularly behavioral addictions, appear at startlingly young ages (Kuss and Griffiths, 2012; Tomei et al., 2021). Although there is ongoing debate over classifying Compulsive Sexual Behavior Disorder (ICD-11) as a behavioral addiction (Kraus et al., 2016; Sassover and Weinstein, 2022), for the purposes of this discussion, these behaviors are characterized as addictive due to shared criteria and the functioning of coping modes. Moreover, as mentioned earlier, these behaviors can and do manifest in childhood.

*e.g. ‘I can remember trying to talk to my dad about a fight with a friend at school when I was in fifth grade. He told me I did not need that friend anyway and to stop thinking about it. So, I went in my room and masturbated and felt better.’ adult sex and alcohol abuse*

*Escapist and self-soothing behaviors* in childhood or adolescence (where the roots of addiction lie) are reinforced and become more frequent over time. These are later joined by secondary *“colluding” behaviors* – concealment, denial, minimization – which enable the individual to hide the evolving addiction, avoid criticism, and evade potential curtailment of addictive behaviors which have become more and more central to their lives (Endrass et al., 2018; Liu and Ma, 2018). Over time, thickening intrapsychic walls develop between the individual’s general self-image and their addictive selves; this pattern of “living two lives” makes healthy attachment even more difficult to attain. Loneliness, shame, rejection, and hopelessness increase and fuel the burgeoning addiction (Leach and Kranzler, 2013).

The colluding behaviors that develop as enablers of the addiction are often as entrenched as the addiction itself, becoming intrapsychic barriers to accessing treatment (Moeller et al., 2022). Harsh inner critics (“you are hopeless”), high levels of detachment, and deception of self and others become standard (Luoma et al., 2012). With time, more intensified webs of deceit and avoidance surround the escalating addictive behaviors, which are extremely difficult for the patient to acknowledge publicly (Pickard, 2016). Webs of avoidance and fears of giving up the addiction make it very difficult for patients to admit to needing help (Luoma et al., 2012).

It is critical to note here that problems with early attachment often underlie dysfunctional personality development (Herbers et al., 2014; Miljkovitch et al., 2018; Fraley and Shaver, 2021) alongside addiction. Personality disorder symptoms, (e.g., affective instability, impulsivity) strongly contribute to the thinking and behaviors which “support” addiction. For example, in a large longitudinal study, Thomas et al. (1999) found that one year following treatment, 94% of patients with personality disorders relapsed vs. 56% of those without such disorders (Newton-Howes et al, 2017; Foulds et al., 2018). This suggests that sustained benefits in treating addictive disorders may require models that attend to both attachment *and* personality (Miljkovitch et al., 2018; Fraley and Shaver, 2021).

Post-Traumatic Stress Disorder (PTSD) also plays a role in complicating treatment for addictions (Najavitis et al., 1997; Brady et al., 2004). One large study estimated that if you change comorbidity from PTSD and addiction to interpersonal trauma (sustaining an injury to attachment system) and addiction, the prevalence rates go from highs of 59% to highs of 99% (Najavitis et al., 1997; Padykula and Conklin, 2010). Treatment approaches that incorporate healing a patient’s interpersonal trauma are needed (Padykula and Conklin, 2010; Tapia et al., 2018).

## 2 What’s currently available

Current Substance Use Disorders (SUD) and behavioral addiction treatment programs include detoxification, medically managed withdrawal, outpatient (short or long-term), intensive outpatient, individual counseling, groups and short- and longer-term residential facilities (NIDA, 2020a,b). Most of these were originally designed as modifications of the 12-step treatment approach, but have incorporated coping strategies, tools, and other behavioral interventions (Hayes et al., 2004; NIDA, 2020a). Psychopharmacological interventions (e.g., buprenorphine, naltrexone, methadone have also been used on their own or with some counseling (Ali et al., 2017). However, medication alone does not address the root psychological issues driving addictive behaviors (Zajac et al, 2017).

Patients meeting DSM criteria for PTSD may be offered treatment in designated “trauma tracks” such as Eye Movement Desensitization Reprocessing or trauma-informed group therapy (Rosen et al, 2002). Although these treatments show promise for some patients, these often do not address the needs of populations with high comorbidities of more complex mental health issues, requiring more research (Glasner-Edwards and Rawson, 2010; Padykula and Conklin, 2010; Markus and Hornsveld, 2017). They are also not offered to many people who have suffered other types of attachment difficulties or personality problems who may benefit from more integrative treatment.

In recent decades, other treatment models that emerged outside the 12-step tradition have shown promise in treating addictive disorders. The prominent Transtheoretical Model of Behavior Change (TTM), developed in the 1970’s, gained momentum through the 90’s (Prochaska and DiClemente, 1982, 2005; LaMorte, 2019); more recently, Acceptance and Commitment Therapy (ACT) has also been well-received (Luoma et al., 2012; González-Menéndez et al., 2014; Bahrami and Asghari, 2017). Cognitive Behavioral Therapy, Dialectical Behavioral Therapy and Contingency Management have also shown great promise but may not address the needs of patients with comorbid personality disorders well enough for improved outcomes (Davis et al., 2016; Zamboni et al., 2021; Boog et al., 2023). These models address behavior change, skills, and emotion tolerance; however, they are often applied as pieces within more comprehensive, traditional treatment. They also may not address attachment and personality disorders sufficiently to effect lasting change.

For decades, treatment providers have hoped that taking part in intensive 30, 60, or 90-day programs followed by outpatient treatment and community support would lead to long-term recovery for most people (NIDA, 2020a,b). Yet despite their positive short term outcomes (including safety, medical stabilization, connection to outside resources, and greater illness awareness) many treatment models have been largely unsuccessful in sustaining recovery (Ball et al., 2006; Brorson et al., 2013), which seems to require a more holistic or comprehensive approach (Ball, 1998; Kellogg and Tatarsky, 2012). It is estimated as recently as 2020 that treatment “failure” or relapse (defined as use of the same substance or process at the same level and intensity as before treatment) during the first 30 to 180 days in treatment, ranges from 40 to 60 percent (NIDA, 2020a,b). Those relapse numbers remain stable at five years (McLellan et al., 2000; Weisner et al., 2003; Scott et al., 2011). Among patients with comorbid personality disorder, the relapse rate is staggeringly higher (e.g., 94%; Thomas et al., 1999; 90%+ Staiger et al., 2008). We owe it to our patients to do better.

Patients’ engagement in treatment can be adversely affected by therapists’ attitudes toward patients with addictive disorders (Forman et al., 2001). In a study of 317 staff members across 50 addiction treatment programs (Forman et al., 2001), more than a third of those polled expressed critical and even punitive attitudes towards their patients (e.g., wanting more confrontation and believing that noncompliant patients should be discharged) (Crapanzano et al, 2019). This stands in stark contrast to the treatment of other disorders where noncompliance and relapse are accepted and addressed as part of chronic, long-term illness and care (NIDA, 2020a,b).

*“I am tired of the judgement and being told I am not working hard enough. I’m not going back.”*

*four months into treatment after his second relapse.*

In an extensive systematic review of relapse factors in the treatment of addiction, which included 122 studies and almost 200,000 patients, Brorson et al. (2013) reported finding an overwhelming focus (96%) on patient factors rather than treatment type in the prediction of outcomes. This is aligned with a traditional medical model of addiction, which views negative outcomes (e.g., drop-out) as the exclusive result of underlying patient psychopathology (Brorson et al., 2013). As Brorson and her colleagues note, what is missing from most studies was any focus on the dynamics between patients and the type of treatment they received (McKellar et al., 2006; Brorson et al., 2013). In one study that did explore these issues, high risk patients (e.g., younger ones, or ones suffering from cognitive deficits) were less likely to drop-out when the treatment environment was appraised as high in support, i.e., when attachment needs were sufficiently met (McKellar et al., 2006). Similarly, Ball et al. (2006) found that one of the top two reasons for dropout was conflict with staff (Moyers and Miller, 2013).

I argue that the primary emphasis of many treatment modalities on behavioral and cognitive interventions leaves much to be desired given comorbid issues of personality and attachment. Patients often cannot attach or remain attached in community support groups or treatment programs; do not share core values with popular support groups; or otherwise, do not remain engaged (Ball et al., 2006; Dingle et al., 2018; Zilberman and Messer, 2020). A more comprehensive, transdiagnostic approach is needed to address the multifactorial nature of addictive disorders (Ball, 1998; Glasner-Edwards and Rawson, 2010; Boog et al., 2018). I propose Schema Therapy as an alternative, evidence-based (for complex, persistent diagnoses), and more integrated approach to treatment.

## 3 Schema therapy: what it has to offer

Schema Therapy (ST) is well positioned to treat addictive disorders comorbid with attachment and personality problems (Sempértegui et al., 2013). It has a substantial evidence base for treating patients with maladaptive behaviors, personality disorders, or complex trauma holistically (Sempértegui et al., 2013; Bamelis et al., 2014; Arntz, 2015; Arntz and Simpson, 2020). It can even address what used to be seen as a “habit” driven by cues or emotional pain, as theorized by Boog et al. (2023) (Boog and Tibbeoel, 2023). It is designed to create safe attachment, more securely engaging patients in a comprehensive treatment process which harnesses corrective emotional experiences to achieve sustained change (Rafaeli et al., 2011) – elements that current addiction treatment models lack (Boog et al., 2018).

Important to note, Samuel Ball, Ph. D., conducted research in Dual-Focused Schema Therapy for addictive disorders in the late 1990’s with mixed but promising results (Ball, 1998). At the time of Ball’s publication, the more empirically supported mode model had not yet been formally introduced, which may partially account for the less-than-optimal results with this population (see “Modes” below) (Ball et al., 2006). Lee and Arntz (2013) criticized this study for its high dropout rates and brief therapy duration (24 sessions), which is less than ideal for a study or a complex population (Arntz and Jacob, 2013; Arntz et al., 2015; Arntz and Simpson, 2020; Gonzalez-Castro and Uluğ, 2022). Addictive disorders and personality disorders arguably require sustained, longer-term treatments to achieve positive outcomes.

Schema Therapy is an intentionally crafted, integrative, evidence-based model developed by Jeffrey Young, PhD, and has continued to evolve into its current practice with major contributions to its mode model by Dr. Young and other researchers and clinicians (Rafaeli et al., 2015; Roediger et al., 2018; Heath and Startup, 2020). It is crafted with core elements of object relations theory, gestalt therapy, third wave CBT interventions and others. It focuses on identifying and modifying early maladaptive schemas (EMS), which are self-defeating emotional and cognitive patterns established from childhood experiences and early interactions with others. These schemas can lead to negative thoughts, feelings, and behaviors throughout one’s life. Schema therapy aims to help individuals understand their schemas, learn to recognize and change maladaptive coping styles, and develop healthier patterns of thinking and behaving (Young et al., 2006). Comprehensive case conceptualizations are formulated from detailed assessments; emotion-focused, cognitive, and behavioral interventions are used to address problematic behavioral patterns (modes) that result from faulty, emotional perceptions, and interpretations of themselves and the world (schemas) (Roediger et al., 2018). Attachment, issues of personality, and dysfunctional behavioral coping modes are central features of this model which make it very promising for the treatment necessary for the problems facing people with addictions. It is a transdiagnostic approach to treatment that has demonstrated positive outcomes with populations that have historically used addictive modes as methods of coping with life. e.g., Personality Disorders (Sempértegui et al., 2013; Arntz et al., 2022; Boog et al., 2023).

In studies with personality disordered patients who are traditionally difficult to retain in treatment, ST has been able to keep patients engaged longer than other treatment models (Sempértegui et al., 2013; Arntz et al., 2015). The model’s use of the therapy relationship as a vehicle to meet core needs and implement evidence-based interventions makes it a promising model for this population (Sempértegui et al., 2013). *The overarching goals of schema therapy are to meet core developmental needs; heal dysfunctional schemas; and help the patient strengthen and use their own healthy adult mode over the dysfunctional coping modes to which they default.* Addiction is conceptualized in ST as a dysfunctional coping mode: dysfunctional behaviors, emotions and thinking to a significant degree (Thalmayer and Andreas, 2022).

Young’s definition of “schema” is that a schema is a pattern imposed from their experience to help individuals to explain it, to mediate perception and to guide their responses (Young et al., 2006; Baião et al., 2021; Thalmayer and Andreas, 2022). It is a broad organizing principle which makes sense of one’s own life experience. It contains emotions, sensations, and thoughts (Young et al., 2006). Schemas can be positive or negative. ST is concerned with Early Maladaptive Schemas (EMS) which are schemas that are dysfunctional to a significant degree. They are developed in childhood and adolescence through the frustration of core developmental needs integrated with innate temperament (Young et al., 2006; Aloi et al, 2020). Young defines core needs as: secure attachment (safety, stability, nurturance); autonomy (sense of identity); freedom to express valid needs and emotions; spontaneity and play; and realistic limits and self-control (Young et al., 2006).

According to Young, and others, the pain of these EMS’s lead to the development of self-states or schema modes (Rafaeli et al., 2011; Arntz and Jacob, 2013). These modes are split off parts of the self, unconscious to a larger or lesser degree, depending on childhood experiences, temperament, and other factors (Rafaeli et al., 2015). Child Modes, Maladaptive Coping Modes (MCMs) and Inner Critic or Internalized Parent Modes, along with Healthy Adult Modes, are the broad categories conceptualized in the schema model (Arntz and Jacob, 2013; Edwards, 2022).

Child modes are thought of as underdeveloped, “young” parts of the patient that remain undeveloped after the patient failed to sufficiently have their early core needs met (Rafaeli et al., 2015). These modes are often hidden by addictive modes since addiction masks the pain associated with them (Lazarus and Rafaeli, 2021). Child modes can often be described by the pain experienced by this undeveloped self, including lonely child, angry child, abused or abandoned child. Impulsive or undisciplined child modes can develop when the child or adolescent has had overly strict or permissive discipline or limits imposed by care givers (Rafaeli et al., 2015). The pain in these modes is conceptualized here as the activators of the addictive mode cycle (Boog et al., 2018).

As a result of the distressing negative emotions, sensations, impulses, memories, and cognitions activated by EMS concordant with the patient’s coping styles, coping modes develop (Young et al., 2006). These long-standing, often unrecognized, cognitive, affective, interpersonal, and behavioral responses to the triggering of schemas are called *maladaptive coping modes (MCMs)* (Young et al., 2006). Although these MCMs may effectively regulate the negative affect associated with schema activation in the short term, they are self-defeating and impede the meeting of core needs and the change process (Young and Klosko, 1994; Young et al., 2006; Heath and Startup, 2020). In the long term they are schema reinforcing (Young et al., 2006; Fuhr and Gondara, 2021; Edwards, 2022). Such is the case in an addictive mode.

MCMs are categorized through the three styles of coping: schema surrender, schema avoidance, or schema overcompensation (Edwards et al., 2022). Schema surrender represents a complying or giving into the schema, for example, giving up and surrendering to the idea that you will always be defective so “why try” (Young et al., 2006; Edwards et al., 2022). Schema avoidance or detachment includes various forms of escape or avoidance from the schema or situations that activate the schema, for example, social withdrawal, excessive autonomy, compulsive stimulation-seeking, compulsive self-soothing or thrill-seeking, and psychological withdrawal (Young et al., 2006; Ball et al., 2006). Schema overcompensation involves different forms of “doing or acting the opposite” or countering the schema, for example, aggression or hostility against perceived criticism; excessive self-assertion, recognition, or status-seeking countering feelings of social isolation; manipulation, passive-aggressive rebellion against feeling trapped; and excessive orderliness and control against feelings of vulnerability (Young and Klosko, 1994; Young et al., 2006; Rafaeli et al., 2015). Addictive behaviors would be considered avoidant and detached protector coping modes (Boog et al., 2018).

Lastly, there are internalized modes, such as harsh or demanding inner critics or internalized parent modes (Young et al., 2006; Vîslă et al., 2022). These are thought of by Young as internalized representations of the caregivers, other adults, or the environment. When mobilized, these inner critic modes tend to be schema activating, causing a cycle of dysfunctional coping responses followed by more harsh judgement (Pickering et al., 2018). The individual who has not yet developed methods of coping through their own healthy adult mode is left in cycles of pain, poor coping responses, self-criticism and then more pain (Offergeld et al., 2021; McCarthy et al., 2022).

Schema Therapy utilizes cognitive, behavioral and emotion focused interventions to achieve its goals of meeting core developmental needs in the patient’s current life; achieving healthy attachments; minimizing use of dysfunctional coping modes; and the strengthening the patient’s healthy adult mode (Rafaeli et al., 2015; Thalmayer and Andreas, 2022). The use of empirically proven interventions such as imagery rescripting, cognitive restructuring, psychoeducation, behavioral exposure, empathic confrontation of dysfunctional modes, chair work and the like are employed (Arntz, 2015; Behary, 2020; Thalmayer and Andreas, 2022).

### 3.1 Addictive schema mode development

#### 3.1.1 Triple mode cycle and colluding coping modes: proposed conceptual mode model

**Figure 1:**
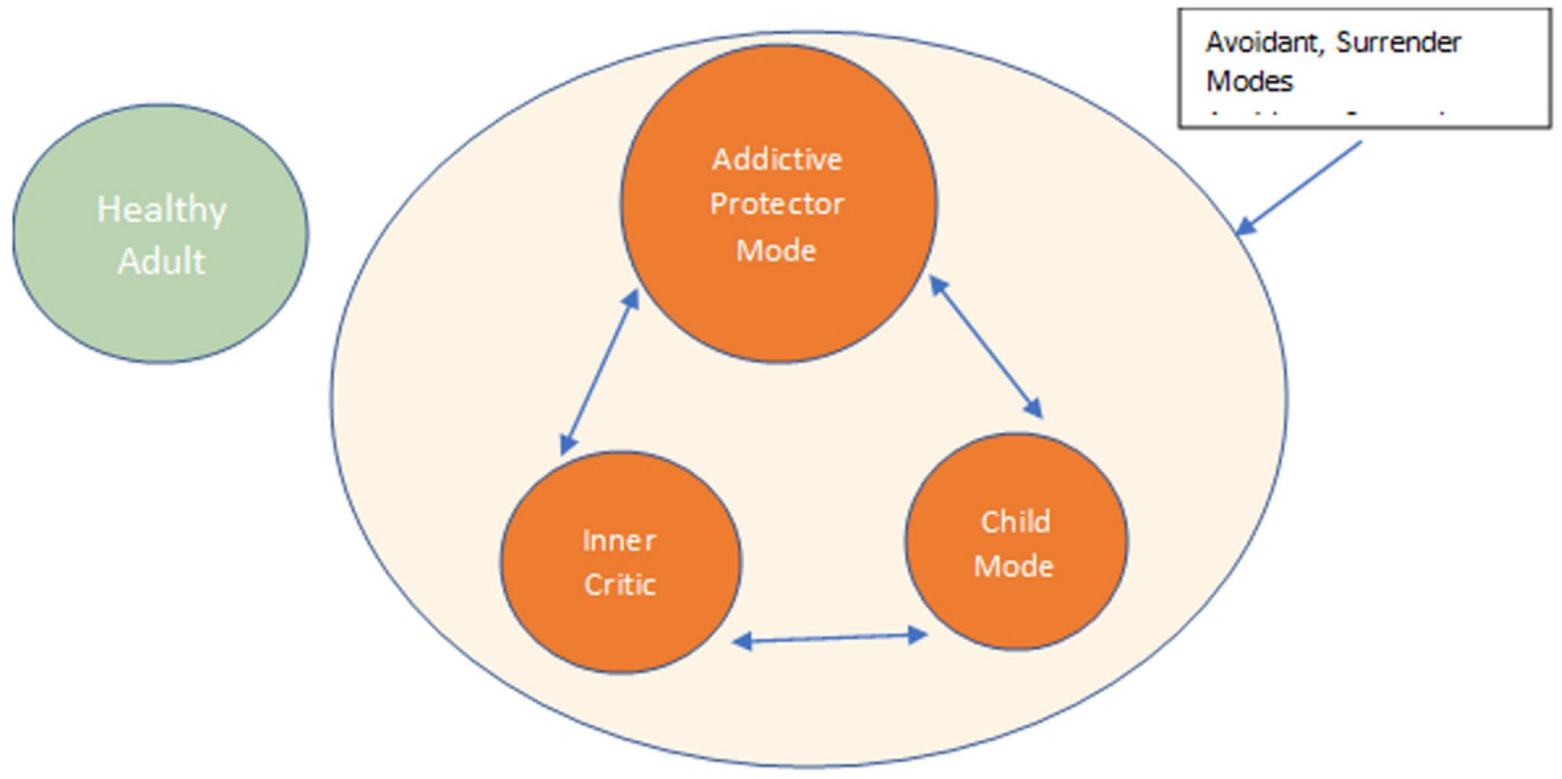
IC: inner critic; SSAPM: self-soothing addictive protector; VC: vulnerable child; HA: healthy adult.

The pain of EMSs formed by the frustration of core needs leads to the development of addictive protector modes (Boog et al., 2018). Importantly, these protector modes serve as one of three points in a Triple Mode Cycle (TMC) which is present at the core of all addictive disorders. Specifically, this TMC is comprised of a Child Mode, an Addictive Protector Mode (APM), and an Internalized Critic Mode. See Figure 1.

Child Modes are aspects of the individual that have not had core needs met and therefore have not matured, still suffering the pain of childhood. When EMSs are triggered, this ensuing pain is felt by the Child Mode, who calls on the APM for its familiar behavior or substance to regulate negative affect, and thus numb, distract, or avoid feelings (Young et al., 2006).

In this conceptualization, I propose that APMs develop as a way of coping with the pain associated with the frustration of the emotion regulation system, a result of deficient attachment, and the activation of EMSs (see Table 1 for common APMs). When individuals lack robust primary attachment systems to manage distress, they must find other means to accomplish this regulation (Sakiris and Berle, 2019; Ein-Dor et al., 2021). These could be defined by their environment, behavior, or substance within reach (perhaps modeled) that appears to satisfy their core needs to a certain extent (Young et al., 2006). In childhood this might manifest as lengthy periods of fantasy, solitary play, or reading books to seek solace. As the child transitions into adolescence and adulthood, they turn to more expedient or mature behaviors. Just like other disorders that use avoidance techniques for coping (i.e., anxiety disorders, binge eating disorders), the behavior or substance proves initially quite positively reinforcing, thereby encouraging use (Simpson et al., 2010).

**Table 1:** Types of addictive protector modes.

Preoccupied, impulsive protector mode	(e.g., drug-seeking, pornography, internet, gaming).
Thrill-seeking protector mode	(e.g., fetishes, illicit activity, “freedom” seeking, risky or stimulating drugs, gambling).
Self-aggrandizing protector mode	(e.g., big daddy sexual fantasies, affair heroes, gamblers, shopping, fantasy play).
Self-soothing protector mode	(e.g., pornography, food, shopping, alcohol, benzodiazepines, opiates).

The APM is often so effective in the short-term, that it starts to dominate more and more thoughts, behaviors, and emotions. After a period of employing this mode, the individual develops more feelings of defectiveness, shame, and guilt. This can occur due to self-judgement, or result from the impact of these behaviors, such as poor work performance, relationships, and personal goal attainment. Patients who already harbor a powerful inner critic will find this mode reinforces their negative feelings (Rafaeli et al., 2015). As this inner critic intensifies, it reactivates the vulnerable child mode (Baião et al., 2021).

*Dave built models as a 5-year-old, read for hours alone as a 10-year-old then later stared at posters of Farrah Faucet on his wall all while his parents fought bitterly in the other room. Later, after years of high anxiety and low self-confidence with girls, he found “kind” sex workers and gained confidence, soothing and control. Later, feeling terrible guilt and fear of being found out.*

As the addiction develops, it strengthens. Families, communities, and even the addicts themselves may see these behaviors as irrational, chaotic, and self-destructive to a significant extent, resulting in the frustration of core needs, especially healthy attachment (Leach and Kranzler, 2013). They often fail to recognize that these behaviors, as dysfunctional as they may be, served a function. Helping the patient comprehend this is a critical component of sustained therapeutic engagement, as it diminishes the influence of the inner critic, thereby reducing feelings of shame and behavioral avoidance (Green and Balfour, 2020).

Over time, the patient begins to hide the TMC from others and *from themselves*. This “hiding” spawns the Avoidant Colluding Mode (Endrass et al., 2018; Liu and Ma, 2018), whose function is to avoid (inner and outer) criticism and thus, allow the addictive behavior to continue. Avoidant Modes work to maintain the intrapsychic wall that separates the addiction from the rest of the patient’s life. They create strong denial and a fragmented sense of self, through minimizing, lying, missing therapy sessions, or other avoidant behaviors.

Another common Colluding Mode employed by addicted patients is the Compliant Surrenderer (Bai et al., 2019), which supports the addictive behavior by reinforcing maladaptive schemas such as emotional deprivation, mistrust/abuse, negativity, and insufficient self-control. In this mode, the patient will feel hopeless, demoralized, and helpless to change (Rafaeli et al., 2015). They will develop negative thinking patterns that reinforce ideas such as “it will always be this way,” “there is no hope”; these patterns ensure that the patient continue to see the addictive behavior as their only means of coping with distress.

These secondary avoidant and colluding modes need to be addressed by the therapist and understood by the patient to effectively remain engaged and progress in treatment. Breaking down the wall and helping the patient become fully aware of their entire self and the reality of their compartmentalized life becomes a critical clinical role for the therapist (VanVresswijk, 2020). Through the clinician’s interventions, the patient can start to develop the inner motivation to work toward abstinence from destructive behaviors (See interventions).

The patient’s Healthy Adult Mode will be engaged to assist in meeting the patient’s core needs, healing schemas and shifting out of dysfunctional modes. However, patients will vary widely in the strength and sturdiness of their Healthy Adult at the outset of treatment (Edwards et al., 2022; Thalmayer and Andreas, 2022). This mode’s strength will depend on a patient’s history. The therapist will need to fill in for the patient’s Healthy Adult Mode to a lesser or greater extent depending on its strength (Thalmayer and Andreas, 2022). At the start of treatment, this mode is usually experienced by the patient as weak (Edwards et al., 2022). Strengthening it is a central focus in ST, allowing the patient to become independent and stable, achieving comprehensive, sustained recovery and obviating the need for the therapist to fill in (Rafaeli et al., 2015).

### 3.2 Schema therapy for addiction treatment (stat): a proposal

#### 3.2.1 The therapy relationship: a foundation for treatment

*“When my addicted patients look at me, they are looking for the real me...they gauge with unerring eyes whether I am grounded enough to coexist with them, to listen to them.”*

Gabor Mate, MD, p. 25, In the Realm of Hungry Ghosts.

The initial work from the first contact is establishing the bond between the primary clinician and the patient (VanVresswijk, 2020). The therapeutic relationship is the foundation that makes treatment retention and intervention possible. I suggest a stance of radical, therapeutically appropriate authenticity and equality with the patient (VanVresswijk, 2020). Trust is a critical element in treating patients with addictions, as they have frequently been treated with disdain from their family, community, and inner critic, and at times have felt rejected by treatment providers (Green and Balfour, 2020).

The clinician must always keep in mind the conceptualization of addictive disorders so they can help convey and directly talk about how “this is understandable and changeable,” that it all makes sense in context (Dingle et al., 2018; Green and Balfour, 2020). This is especially important because patients will likely not understand their own behaviors well, and neither will people close to them. Both patients and their community may consider the patients to be “crazy” or “out of control,” as they engage in behaviors that often seriously hamper any change of stable, desirable existence. The patient’s inner critic which degrades them, creates even more barriers to recovery. Keeping the conceptualization in mind and linking it to present behaviors will help defuse dysfunctional self-critical modes and allow patients to understand their own mode cycles for themselves (Dingle et al., 2018; Green and Balfour, 2020).

The therapeutic relationship is the cornerstone of all interventions with the patient from the first call through termination (Levy et al., 2018; VanVresswijk, 2020). Through the relationship with the patient, you will be able to meet core emotional needs at every stage of therapy. Sometimes directly, through the expression and modeling of connection and care for the client struggling with pain and destructive behavior and sometimes indirectly through specific therapeutic interventions (see below). A strong therapeutic relationship (and later, a healthy relationship of patients to themselves) is one in which support, caring, direction, and limit setting can occur without rejection, enmeshment, or detachment (Skewes et al., 2015). The case conceptualization will be the roadmap that identifies the patient-specific needs necessary for the patient to become more integrated, strengthen their healthy adult mode, heal old schemas, and stop using substances or processes compulsively.

The therapist will more directly function as the patient’s healthy adult self at the beginning of treatment thereby providing a type of limited reparenting and healthy role modelling (Arntz and Jacob, 2013). As the therapy progresses and the patient’s healthy adult mode becomes sturdier, the therapist takes less of a role in this function. The extent to which this is necessary will depend on the case conceptualization. Clinicians must use flexibility within each session: they must balance empathic confrontative approaches with limited reparenting that meets core needs and heals painful schemas (Skewes et al., 2015; Behary, 2020), and behavior change techniques with emotion-focused approaches.

As the clinician slowly meet and help the patient experience their needs being met, attachment grows between them. Most importantly, the patient *experiences* what it like to have their needs met in the context of a healthy relationship (Shaver and Mikulincer, 2007; Levy et al., 2018). The *experience* is critical and can be achieved during all interventions. Often patients have been “attached” to their addictive behaviors for so long, they do not know what a true healthy attachment is like. It is necessary for them to know what it is like, so the patient learns to have healthy relationships outside of the therapy room. If we accept that attachment is at the center of addictive disorders, then this is crucial for sustained recovery (Zapf et al., 2008).

### 3.3 The ST assessment phase

In ST, assessment is used to develop a case conceptualization, which serves as the roadmap for treatment (Green and Balfour, 2020). We use evidence-based tools such as the Multimodal Life Inventory (Young and Klosko, 1994) and a thorough biopsychosocial assessment interview. The Schema Mode Inventory (SMI) (Young et al., 2006; van Vreeswijk et al., 2015) and Young Schema Questionnaire (YSQ-L; Young and Klosko, 1994; Yalcin et al., 2023) should also be employed (Young et al., 2006). Assessment helps flesh out intricate details of early frustration of core needs and attachment ruptures, the patient’s first detachment episodes, any family history of addictive behaviors, modeling of detachment, or avoidance of emotions, and of course the history of the addictive behavior itself: its inception as well as its progression, year to year or event to event (Young et al., 2006).

Depending on the nature of the addictive disorder(s), clinicians will also use empirically supported tools specific to the patient’s addiction. Thus, tools such as the TAPS Inventory (Tobacco, Alcohol, Prescription Medication, and other Substances), CSBI-13 (Compulsive Sexual Behaviors) or BBGS (Brief Biosocial Gambling Screen) would be applied as needed (Santos et al, 2017; Andreassen et al., 2018; Pickering et al, 2018). Clinicians should be sure to obtain details of the rituals involved before and after the core addictive behavior occurs. This will provide important indications of the subtle initial “adaptive” function of the addictive mode and its colluders (Young et al., 2006). It will also outline the formation of the TMC (Rafaeli et al., 2015; Farrell and Shaw, 2020). The original function – typically, an attempt to meet core needs – may include distraction from boredom or loneliness, freedom-seeking, increasing self-esteem and/or social standing, pain avoidance, and the like (Young et al., 2006; Santos et al, 2017; Aloi et al, 2020). Family members can and should be included in the assessment process where the addiction is known to them, and the patient is willing (Velleman et al, 2005; Young et al., 2006). This will provide a more holistic assessment and may, in some cases, stabilize the patient at home during preliminary treatment (Velleman et al, 2005; Young et al., 2006).

Addictions certainly can and do run on their own steam over time (McLellan et al., 2000). Physical dependence, cognitive impairment due to addiction and lives that have been built around the addiction are all part of fueling the cycle along with poorly functioning coping modes (Robertson et al., 2021). This can make assessment complex (Brorson et al., 2013) Behavior chain analyses or functional analyses (McHugh et al, 2010; Rizvi and Ritschel, 2014) of relapse will help determine the function of the modes and the multifactorial relapse pattern (Fatseas et al., 2015).

The clinician will need to determine the strength of the patient’s Healthy Adult Mode (HAM), which can often be determined through the patient’s history, current functioning level, duration and intensity of the addiction(s) (Gonzalez-Castro and Uluğ, 2022). A patient with an addictive cycle that began early in life and is highly entrenched will have a weaker healthy adult mode than one whose addiction emerged later in life and is more intermittent (Boog et al., 2018). Knowing how strong and how accessible the HAM is for a patient will help the clinician decide when and to what extent they need to “step in” to aid or bolster the patient’s healthy responses through modeling, education, reparenting, and/or healthy limit-setting, until the patient can do so for themselves (Rafaeli et al., 2015).

**Figure 2:**
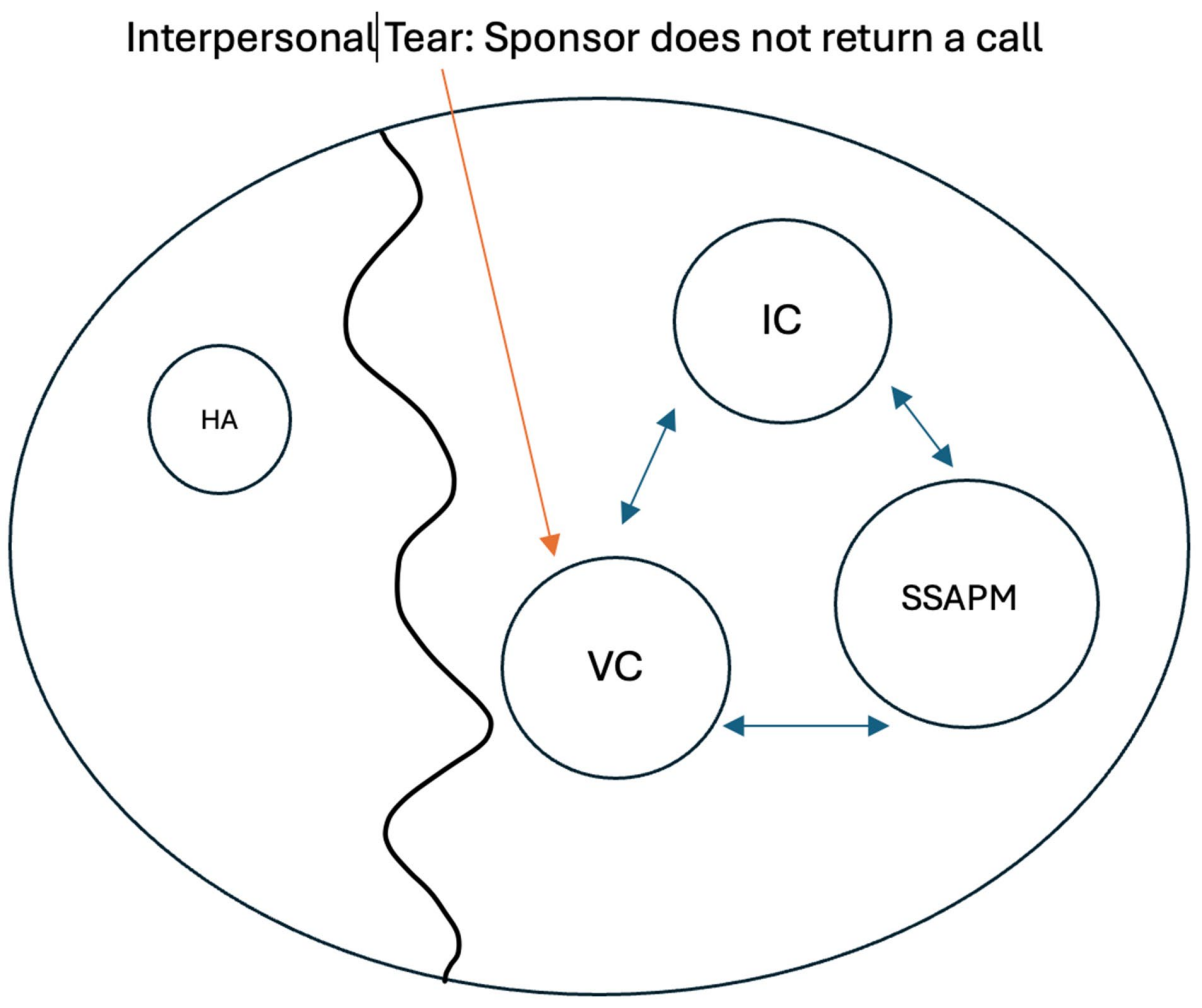
Mode Map Example IC: inner critic; SSAPM: self-soothing addictive protector; VC: vulnerable child; HA: healthy adult.

The assessment phase will require at least one but often three or more appointments so that enough information can be gathered to formulate a case conceptualization and useful mode map which captures, as accurately as possible, the dynamics between the healthy adult, the TMC, and the colluding modes (see Figure 2) (Simeone-DiFrancesco et al., 2015). Once that map is in place, the therapist should be able to tell the story of the patients’ (often valiant) attempts to get their core needs met in ways that invariably have some short-term benefits (e.g., social contact, distraction from boredom and loneliness, excitement, boosts in feeling of self-worth, reductions in pain) but do not meet their underlying core needs or longer-term goals (Khantzian, 2003; Young et al., 2006).

*Activating event: Sponsor does not return a call. Attachment tear occurs intrapsychically and triggers old feelings of emotional deprivation(schema). Patient surrenders to the schema activation and surrenders to the message “no one will ever care for me” (VC), concluding ‘I need the addiction to alleviate the pain, there is nothing else’. The patient’s healthy adult mode (HA) is too small and remote to counter this pain and conclusion. The patient uses the addiction once again (SSAPM), feeling self-critical later for having given in (IC), feeling hopeless that this can change. Cycle occurs largely with no connection to an internalized healthy adult*

The assessment phase helps determine whether STAT can begin safely, or instead, whether the patient first requires some stabilization prior to it. If the latter is the case – e.g., if a patient is consuming so much alcohol that unsupervised detoxification could endanger their health, or if their sexually compulsive behavior cannot be stopped or limited and puts them or others at imminent risk – the clinician may recommend a short-term in-patient stay (Carew and Comiskey, 2018; George et al., 2018).

### 3.4 The stat treatment phase

#### 3.4.1 Balancing each session: an overview

By the time the therapy advances to the treatment phase, the clinician would have developed a comprehensive case conceptualization and mode map (Edwards, 2022). This important groundwork forms the basis for planning treatment, setting both short-term and long-term goals for behavioral change, building and strengthening the patient’s HAM, and establishing the support system necessary to facilitate these changes (Young et al., 2006; Glasner-Edwards and Rawson, 2010). The case conceptualization also helps clinicians prioritize alternative foci within specific sessions (Green and Balfour, 2020). Through this conceptualization, clinicians are advantaged in maintaining a balance between interventions aimed at behavioral change, empathic confrontation of coping modes, emotion-focused work, or simply addressing unmet needs directly (Young et al., 2006; Rafaeli et al., 2011).

The beginning of treatment, whether the long-term goals are harm reduction or abstinence, should prioritize simple behavioral change. This initial phase is crucial as it lays a stabilizing foundation for subsequent steps in the recovery process (Carnes, 1998; Kellogg and Tatarsky, 2012). One commonly used tool during this phase is behavioral chain analysis, which helps identify the sequence of events (including maladaptive coping modes) leading to addictive behaviors (McCarthy et al., 2022). Clinicians will address poorly functioning behaviors using a strategy referred to as empathic confrontation, described below, among others. They also work on enhancing decision-making skills that align with recovery goals and encourage patients to build a supportive network of individuals to provide assistance and accountability. This network can serve as a valuable resource in achieving and maintaining stability, especially since healthy attachment is vital (Tracy et al., 2012; Leach and Kranzler, 2013).

This stage of treatment may resemble traditional addiction treatment approaches in some aspects, but there are unique elements that set it apart. The focus on the therapeutic relationship, the incorporation of a case conceptualization that addresses attachment needs, and the use of mode-focused intervention strategies distinguish it from traditional treatment in terms of content, process, and tone (Kellogg and Tatarsky, 2012).

For instance, if a patient fails to attend their therapy group each week, our approach will involve identifying the activated modes and schemas that might have hindered the patient’s progress towards previously agreed-upon behavioral goals. Techniques such as imagery, empathic confrontation of the modes, setting realistic limits, and behavioral rehearsal may be utilized to overcome barriers and facilitate the achievement of these short-term objectives (Farrell and Shaw, 2020).

It is essential to assess the patient’s mode state at the beginning of each session throughout the treatment process to balance sessions accurately (Rafaeli et al., 2011). This assessment allows the clinician to determine the most suitable interventions for the patient’s needs *in vivo* (Young et al., 2006). Patients may enter the session in various modes, such as highly avoidant, surrender, self-critical, or vulnerable modes (Rafaeli et al., 2015). It is crucial to identify these modes, empathically confront them, and work through them to effectively address any obstacles to changing addictive behaviors (Gonzalez-Castro and Uluğ, 2022).

*For example, M appeared calm and agreeable in the session, but he revealed that he had been drinking and engaging in BDSM activities over the past weekend. His passive and compliant attitude indicated that he was likely in a surrender mode. Before addressing his slip and discussing ways to prevent further relapse, I needed to employ interventions to help him shift out of the surrender mode and become more engaged in the therapeutic process (Roediger et al.,* 2018 *).*

The clinician should always spend time exploring relapses, slips, urges to engage in behaviors that the patient was previously motivated to change (Moeller et al., 2022). By exploring these occurrences, the clinician can assist the patient in understanding why they revert to addictive modes and behaviors (Gonzalez-Castro and Uluğ, 2022). Understanding why the patient defaults to these behaviors empathically while aligning against dysfunctional behaviors will help the patient avoid falling into their inner critic, and thus prevent the reactivation of maladaptive schemas subsequent addictive modes (Rafaeli et al., 2011).

In balance, the clinician will employ various techniques such as behavior change strategies, problem-solving skills, mode dialogues incorporating chair work and imagery, and addressing present-day challenges (Maurer and Rafaeli, 2020; Kellogg and Garcia Torres, 2021). These methods aim to directly tackle the barriers preventing the patient from achieving their desired behavior change goals (Gonzalez-Castro and Uluğ, 2022). The remaining portion of the session can be devoted to exploring and addressing any triggered modes, examining attachment dynamics with the therapist or the patient’s support system, and addressing core needs (Flores, 2001; Young et al., 2006).

Sessions will dynamically shift between homing in on specific dysfunctional addiction-related behaviors and adopting a more comprehensive approach to address more holistic patient problems, depending on the active mode or the patient’s treatment progress.

### 3.5 Empathic confrontation

*Patients are doing the best they can, and they need to change...*

Core Principle of Dialectical Behavioral Therapy, Marsha Linehan

Empathic Confrontation is an essential dialectical stance used by therapists to address maladaptive coping modes and associated behaviors. On the one hand, it involves empathy for these modes’ origins, and for the unmet needs which cause the patient pain (and necessitate coping). On the other hand, it involves recognizing and confronting how these modes and behaviors work against the client’s real needs. Empathic confrontation is particularly crucial in working with individuals who struggle with addictive disorders, but requires great skill, as patients may be sensitive to perceived criticism due to their maladaptive schemas, internal critic, and societal stigmas surrounding addiction (Behary, 2020; VanVresswijk, 2020).

When applying Empathic Confrontation, it is important to emphasize empathy and genuine concern for the patient’s well-being before turning to confrontation. Without such a balance between empathy and setting limits, or without *authentic* empathy, the patient may resort to avoidance strategies, making it more challenging to effectively address the central addictive mode cycle (VanVresswijk, 2020). For example, the clinican can acknowledge and validate the short-term benefits of coping behaviors such as feeling thrilled, soothed, compensated, or emotionally numbed. This helps the patient feel heard and understood before moving on to confront the behavior in question. Acknowledging these short-term benefits then allows the therapist to address their longer-term harm and the way in which they maintain the addiction – now better understood as a coping mechanism (Young et al., 2006). This approach serves several purposes: it strengthens the therapeutic relationship, reduces the influence of the patient’s inner critic, acknowledges and meets the patient’s core emotional needs for validation and understanding with appropriate boundaries, aligns with the patient’s healthy adult self, and encourages the patient to remain engaged without avoiding confronting their dysfunctional behavior.

It is crucial to address the patient’s internalized critic (or demanding punitive parent mode), whether it emerges during therapy sessions or between them (Pugh, 2018). Patients often believe that their inner critic’s judgments are veridical. It, rather than their healthy adult, is thought to see them for “who they really are”: dysfunctional to the core and morally bankrupt (Rafaeli et al., 2011). Moreover, many patients believe they *need* the inner critic to motivate their behavior change and support their sobriety efforts; in fact, true recovery is usually *hindered* by this mode, which reinforces the TMC and thwarts the patient’s efforts to set achievable limits and gain lasting behavioral changes (Gonzalez-Castro and Uluğ, 2022). The clinician aims to help the patient distinguish between the critic and the Healthy Adult mode, which is much more effective in reducing shame, deactivating maladaptive schemas, and ultimately diminishing reliance on the addictive mode cycle (Edwards et al., 2022).

### 3.6 Behavioral interventions

Throughout the treatment process, therapists will need to utilize a range of behavioral interventions (Glasner-Edwards and Rawson, 2010). Initially, therapists will need to take a more directive approach, as the patient’s ability to engage in healthy coping mode responses will be underdeveloped and the inclination to rely on destabilizing modes may be pronounced (Arntz and Jacob, 2013). Repetition and rehearsal of these interventions will be necessary throughout treatment, with a particular focus in the early stages (Kellogg and Tatarsky, 2012).

Behavior Chain Analysis is instrumental for therapists to identify and define target behaviors for change, potential triggering events, and to prevent relapse (Rizvi, 2014). At the beginning of treatment, therapists can utilize this technique to assist patients in identifying events with associated schemas that activate addictive modes between sessions and work together to develop strategies for change that support the patient’s goals(Gonzalez-Castro and Uluğ, 2022). Additionally, Behavior Chain Analysis can help prioritize and target other problematic behaviors (Dimeff and Linehan, 2008). As treatment progresses, this tool can be used to identify areas in the patient’s life that may require additional support or behavior change to maintain progress and prevent relapses.

Behavioral rehearsal is a valuable technique that can assist patients in learning how to effectively cope with day-to-day conflicts and psychosocial issues that arise while working towards their recovery goals. It can be achieved through various methods such as schema mode dialogues (Farrell and Shaw, 2020; Kellogg and Garcia Torres, 2021), imagery exercises, and role plays. Engaging in behavioral rehearsal enables patients to develop and practice new skills, allowing them to navigate new support systems and establish healthy boundaries with previous dysfunctional patterns (Kellogg and Tatarsky, 2012).

In Schema Therapy, the therapist and patient engage in a limited reparenting relationship to facilitate the development of healthier coping responses (Thalmayer and Andreas, 2022). One method used is Schema Flash Cards (SFC), which aim to help patients explore and adopt new behaviors for achieving healthier coping responses or regulating their emotions during the early stages of treatment (Simeone-DiFrancesco et al., 2015). SFCs typically consist of identifying the triggering event with associated negative emotions, the patient’s repeated past behaviors, a reattribution process, and the reinforcement of a healthier adult response (Simeone-DiFrancesco et al., 2015). These flashcards can be provided to the patient in written format or through voice memos from the therapist (Farrell and Shaw, 2020).

Short, simple homework assignments can be used to reinforce treatment goals and help the patient apply concepts from therapy to their daily life (Young et al., 2006; Goslar et al., 2020). These assignments may involve making changes in daily routines, improving sleep habits, completing writing exercises, seeking emotional support, or engaging in behavioral exposure activities to challenge anxiety and other negative emotions (Powers et al., 2010).

Therapeutic interventions that involve behavioral exposure can help patients build resilience and manage difficult emotions (Hayes et al., 2004). These interventions can include gradually exposing patients to anxiety-provoking situations, teaching them coping strategies to tolerate discomfort, and eventually processing traumatic experiences where indicated (Tapia et al., 2018). The ultimate aim is to empower the patient, helping them build resilience and the ability to navigate and cope with challenging emotions and situations (Edwards et al., 2022).

### 3.7 Emotion-focused interventions

#### 3.7.1 Imagery and imagery rescripting

Imagery and Imagery Rescripting are evidence-based tools that can be used effectively throughout the treatment process (Hagenaars and Arntz, 2011). They can be utilized during assessment to gain a deeper understanding of the patient’s early life experiences, schemas, and mode formation (Young et al., 2006; Arntz, Simpson, 2020). To implement these techniques, the patient is encouraged to visualize images of themselves with caregivers from their early life while describing their feelings, needs, and developmental milestones. If the patient has experienced trauma, it is important to proceed slowly, allowing the patient to have control over the early image and incrementally helping the patient experience negative affect (Arntz and Simpson, 2020). It is recommended to begin and end the session with a soothing place image (Roediger et al., 2018).

The therapist should elicit early childhood images related to the use of avoidant behaviors when the patient began to numb or detach from pain during childhood (Miljkovitch et al., 2018). This will aid in identifying the origins of the TMC (Ferrer Muiños, 2020). Exploring imagery can help the therapist and the patient trace all three modes involved in the TMC, including vulnerable feelings, the use of detachment behaviors, and subsequent feelings of guilt or shame (Rafaeli et al., 2015). Gathering this information will assist the therapist in rescripting, healing the original hurts, and meeting the patient’s core needs (Arntz, 2015). It will also provide insight into the patient’s current triggers that lead them to engage in substance or behavioral processes (Fatseas et al., 2015).

Imagery techniques can be used to break down the patient’s intrapsychic wall, as mentioned above. This involves using imagery to combine the fantasy of the addictive life with the patient’s actual life. By doing this, the therapist can help the patient recognize the negative consequences of the addiction and dispel any illusions that it is not harmful or under control (Foulds et al., 2018). For example, the therapist may use imagery to evoke a recent image of the patient’s obsessive gambling at a racetrack, and then bring in the image of the patient’s new boyfriend walking in and witnessing the behavior. This allows the patient to see and feel the impact of the addiction on their real life (Tapia et al., 2018).

As treatment progresses and the patient becomes more capable of experiencing negative emotions, imagery or imagery rescripting may be used as a deeper therapeutic technique (Arntz, 2015). The primary goals at this stage are to address and heal any current interpersonal traumas, facilitate the development of healthy attachments, and help the patient process any trauma that may be triggering the use of addictive modes (Flores, 2001).

In late phases of treatment, imagery or imagery rescripting techniques may also be used to address other maladaptive schemas or dysfunctional modes that have been identified through the assessment process (Arntz and Jacob, 2013). By this stage, patients will have achieved some level of harm reduction or abstinence, allowing them to focus on processing psychosocial stressors that contribute to emotional pain or hinder progress toward life goals (Scott et al., 2011). While the therapist will still assess the patient’s adherence to recovery goals, they will also target other unmet needs in the patient’s life, facilitating a more comprehensive and holistic healing process (Young et al., 2006).

*M had received highly negative messages about his developing sexual feelings during childhood. As early as the age of 10, he would impose punishments on himself, such as reading religious literature instead of attending his baseball practice, whenever he engaged in masturbation to stop himself. During an imagery exercise, we revisited this distressing memory, and with the patient’s permission, I entered the image as a healthy adult attachment figure. I encouraged him to share his pain and inner conflicts and provided him with accurate, sex-positive information. This represented a significant step towards healing the deep-seated pain that underpinned M’s sexually addictive patterns.*

A newer technique called Imagery for Present Day, developed by Eshkol Rafaeli, PhD, and Offer Maurer, PhD, can be effective in helping patients address current conflicts, interpersonal difficulties, and prevent relapse (Maurer and Rafaeli, 2020). In this technique, the therapist guides the patient to identify any thoughts, feelings, and behaviors that may hinder progress towards their goals, such as maintaining abstinence from destructive behaviors, nurturing healthy relationships, and functioning from a healthy adult state. Imagery for Present Day also provides an opportunity for behavioral rehearsal to reinforce desired changes (Kellogg and Garcia Torres, 2021).

### 3.8 Chair work

Chair work is an experiential intervention commonly used in psychotherapy. It involves clients actively engaging in a conversation or role-playing exercise while moving between different chairs to represent various aspects of themselves or conflicting perspectives. Chair work can be effective for processing inner and outer conflicts, as well as reenacting scenes from the past, present, or future (Kellogg and Tatarsky, 2012; Pugh, 2018). This technique has its roots in psychodrama and Gestalt therapy and has been integrated into various therapeutic approaches. Chair work can be adapted to different situations and can be a valuable tool for facilitating change.

Scott Kellogg, PhD, has an effective model which works well with addicted individuals. He uses four core dialogues with a simple chair technique to work with the patient (Kellogg and Garcia Torres, 2021). This differs from other practitioners who may use many chairs and multiple dialog models for all the patient’s modes. Kellogg’s model simplifies the process for the addicted individual, protecting against more detachment and encouraging a deeper experiential change (Kellogg and Tatarsky, 2012). The patient can more powerfully process their ambivalence in choosing new ways of coping with their emotional distress among other conflicts. In STAT, we primarily use the chairs for the Healthy Adult, Vulnerable Child, Addicted Protector, and Inner Critic Modes.

During the initial stages of treatment or the assessment process, therapists can utilize chair dialogues to achieve various therapeutic objectives. These objectives may include helping the patient gain a better understanding of the messages conveyed by the addictive mode (Gonzalez-Castro and Uluğ, 2022); identifying how the inner critic reinforces the addictive behavior patterns; strengthening the patient’s healthy adult mode (Edwards et al., 2022); distinguishing between the inner critic and the healthy adult (patient’s often painfully believe the inner critic is their healthy adult self or that they could never stop using addictive modes without the critic); and providing the vulnerable child mode with core needs from a healthy adult figure (Young et al., 2006). These chair dialogues are an effective tool in facilitating healing and change within the individual.

Chair work can also be valuable for helping patients recognize and experience the colluding modes that keep them from being aware of why they continue to relapse (Rafaeli et al., 2015). These colluding modes, such as minimizing, avoiding, and surrendering, often serve to keep the TMC intact and the intrapsychic wall up. By using distinct chairs to represent these colluding modes and exploring their messages, therapists can help patients gain insight into the ways these parts of themselves may be hindering their progress. This is another important area where the patient’s ambivalence is addressed. Through this process, patients may develop a stronger motivation to change and a deeper experiencing of their healthy adult self, which is crucial for successful, long-term recovery (Tracy et al., 2012).

It is important to note here that at the beginning of treatment, like in imagery rescripting, the therapist may need to demonstrate a healthy adult mode. The demonstration allows the patient to observe and internalize the qualities and behaviors of a healthy adult (Young et al., 2006). Through this modeling process, the patient can begin to develop their own strong and resilient healthy adult mode that can nurture, set limits, and protect them. As the patient’s healthy adult mode becomes stronger, they will be better equipped to take on this role themselves in the therapeutic process and in their everyday lives (Edwards et al., 2022).

As therapy progresses and the patient can experience feelings without resorting to detachment, the therapist can introduce additional chairs. By utilizing these chairs, the patient can explore and express other modes, gaining a better understanding of their influences on behavior and goals (Rafaeli et al., 2015). The therapist also helps the patient clarify consequences, develop a healthier adult self, overcome the inner critic, and address interpersonal challenges to prevent relapse (Pugh, 2018; Gonzalez-Castro and Uluğ, 2022). This approach promotes growth, self-awareness, and lasting recovery.

### 3.9 Support and process groups

Group therapy plays a vital role in the treatment of individuals with addictive disorders by providing a supportive and therapeutic environment for them to address underlying attachment issues, enhance social skills, and reduce feelings of shame (Flores, 2001; Farrell et al., 2009; Moeller et al., 2022). It is essential that group therapy be facilitated by experienced professionals who are well-versed in developmental and interpersonal models for change (Flores, 2001).

For patients with a support system built around their addictive behaviors, group therapy offers a unique opportunity for corrective emotional experiences and the development of new attachment skills (Dingle et al., 2018; Tirandaz and Akbari, 2018). The positive changes and insights gained in group therapy can extend beyond the therapy sessions, impacting the patient’s relationships with their family, friends, and community (Farrell et al., 2009).

By incorporating group therapy into all addiction treatments, individuals are given a platform to heal and grow, enabling them to transition from treatment and lead fulfilling lives that are free from addictive patterns (Fernández-Montalvo et al., 2008; Farrell et al., 2009).

In addition to professional-led support and therapy groups, 12-step groups and other community-based groups can provide valuable support to individuals in their recovery journey. These groups can offer a sense of belonging, connection, and peer support, especially for individuals who may not have access to costly treatment options or who benefit from additional community involvement (White and Miller, 2007).

Active participation in these groups allows patients to develop lasting friendships and find a supportive community that can help them navigate challenges and maintain their recovery. The camaraderie and shared experiences in these groups can promote a sense of belonging and reduce feelings of isolation, which are often struggles for individuals with addictive disorders (Estévez et al., 2017; Dingle et al., 2018; Tomei et al, 2021).

By attending community-based groups and participating in 12-step programs, individuals can receive ongoing support, encouragement, and guidance from their peers. These groups provide a platform for individuals to share their stories, successes, and setbacks, creating a supportive environment that fosters growth and healing (Roncero et al., 2019).

In conjunction with professional-led therapy and treatment, community-based and 12-step groups can be integral parts of an individual’s recovery journey, leading to improved social functioning, increased self-esteem, and a healthier and more fulfilling life.

### 3.10 Relapse prevention: an on-going task

To prevent relapse, early implementation of relapse prevention interventions is crucial as patients make positive changes. These interventions should address issues such as support group relationships, family conflicts, negative emotions, and sobriety challenges. Ideally, they should be implemented within the first few months for maximum effectiveness (Weisner et al., 2003; Lappan et al., 2020).

Research suggests that high numbers of relapses occur following interpersonal conflicts, even in cases of minor attachment ruptures (Sinha, 2012; Leach and Kranzler, 2013). Therefore, it is crucial to assess for conflicts at all stages of treatment. It is important to help patients identify and address any conflict they may have with their therapist, support system, or other areas of their life (Young et al., 2006; Rizvi, 2011). This may involve teaching communication skills, working through underlying schema activation, or helping the patient develop better emotional regulation strategies (Sheppes et al., 2015). By maintaining healthy attachments and support systems, patients have a greater chance of sustaining their progress (Weisner et al., 2003).

As patients reduce their reliance on addictive coping mechanisms, it is important for therapists to anticipate and address the activation of underlying maladaptive schemas (Rafaeli et al., 2011). For example, if the addictive mode was used to calm the inner critic and unrelenting standards schema, reducing the use of the addictive mode can lead to increased feelings and activation of these schemas (Young et al., 2006; Rafaeli et al., 2011). By anticipating these activations, therapists can use schema techniques to help patients identify, normalize, and ultimately heal these old schemas without relapsing into dysfunctional modes. It is important to address these underlying schemas to prevent relapse (Ball, 2007).

The therapist should regularly listen for behavioral and emotional cues that may trigger urges for the patient to engage in addictive behaviors (Fatseas et al., 2015). This can be achieved through asking patients to track their urges or thoughts between therapy sessions. By identifying these cues, therapists can help patients develop strategies to cope with and manage these triggers, ultimately reducing the likelihood of relapse (Gonzalez-Castro and Uluğ, 2022).

A patient’s tolerance for their emotional life will also need to be expanded as the addictive mode cycle lessens in frequency or intensity (Fernandez-Serrano et al, 2010; Payne et al., 2015). Therapists can help patients enhance their emotional tolerance through various techniques such as behavioral exposure, imagery, mode dialogues, or role plays (Hayes et al., 2004). By engaging in these techniques, patients can gradually habituate stronger feelings and learn to regulate them more effectively. This process of strengthening emotional tolerance will contribute to long-term recovery and reduce the risk of relapse (Payne et al., 2015).

Lastly, the process of letting go of the addictive protector mode can involve significant griefwork (Dingle et al., 2018). Therapists need to recognize that addiction becomes deep-rooted in a person’s life, even if it is secretive and destructive. Friends, family, rituals, and familiar experiences associated with addiction will all need to be replaced, integrated, or transformed as part of the recovery process (Neale et al., 2014). Additionally, individuals may have developed an identity around their addiction, such as being “the rebel” or “the cool girl.” Therefore, it is essential for therapists to provide a space for patients to express and process any feelings or concerns they have about transitioning into a new way of living (Tiffany et al., 2012). By addressing these feelings, patients can begin to navigate their grief and develop a healthier and more authentic sense of self.

## 4 Discussion

There is a need for a comprehensive and holistic approach in treating addiction, as current treatment approaches often fall short in helping patients achieve long-term recovery and a fulfilling life. Research over the years has shown that there are challenges in terms of retention in treatment programs, relapse rates, and overall long-term recovery. These issues can be attributed, in part, to the way addictions are conceptualized and the lack of holistic approaches that address the underlying factors driving addictive behaviors. It is crucial for patients to have hope and strive for a meaningful life, rather than just focusing on abstinence. It is time for a change in the way addiction is approached and treated to give patients the best chance at sustained recovery and overall well-being (Kellogg and Tatarsky, 2012; Marchand et al., 2019; Goslar et al., 2020).

A more comprehensive approach is needed to effectively address the chronic emotional pain, engagement challenges, and etiology of addiction in patients. This will involve considering factors such as attachment, emotion regulation, trauma, and personality disorders as part of the holistic conceptualization. By taking these aspects into account, clinicians can work towards better outcomes in terms of stability, long-term recovery, and overall well-being for addicted patients.

While current treatment approaches make efforts to address these issues, they frequently lack a holistic and comprehensive perspective. These approaches tend to be siloed and do not focus enough on the therapy relationship, emotion-focused techniques, attachment and core needs, and a schema modes concept (Thalmayer and Andreas, 2022). By incorporating these core components, we can better address the emotional pain, engagement difficulties, and underlying etiology of addiction for a more effective and comprehensive treatment approach.

This adapted version of Schema Therapy (STAT) shows promise in addressing the conceptualization and necessary interventions for individuals with addictive disorders. Early research suggests that using schema therapy for addictive disorders may lead to positive outcomes, but further study and investigation is needed (Kellogg and Tatarsky, 2012; Gonzalez-Castro and Uluğ, 2022; Thalmayer and Andreas, 2022; Boog et al., 2023). It is our responsibility to explore innovative, propitious approaches that offer the potential for improved, significant and lasting improvements in the lives of our patients struggling with addictions, and STAT presents one such promising approach.

## Data availability statement

The original contributions presented in the study are included in the article/supplementary materials, further inquiries can be directed to the corresponding author/s.

## Ethics statement

Ethical review and approval was not required for the study on human participants in accordance with the local legislation and institutional requirements. Written informed consent from the patients/ participants or patients/participants legal guardian/next of kin was not required to participate in this study in accordance with the national legislation and the institutional requirements. Written informed consent was obtained from the individual(s) for the publication of any potentially identifiable images or data included in this article.

## Author contributions

EL: Conceptualization, Writing – original draft, Writing – review & editing.
